# Supplementary material for: In silico exploration of Serratia sp. BRL41 genome for detecting prodigiosin Biosynthetic Gene Cluster (BGC) and in vitro antimicrobial activity assessment of secreted prodigiosin
Source: PLoS One. 2023 Nov 15;18(11):e0294054. doi: 10.1371/journal.pone.0294054 (PMC10651056; doi:10.1371/journal.pone.0294054)
Supplement: Attachment — Submitted filename: Response to Reviewers.docx [file pone.0294054.s001.docx]

# **Response to Reviewers**

## **Manuscript PONE-D-23-01982**

On behalf of coauthors, I would like to thanks the academic editor and reviewer who gave detailed and constructive comments on the manuscript, PONE-D-23-01982. We have addressed all the comments raised by the journal and reviewer and revised the manuscript accordingly. Please find in this disposition our changes and detailed explanations for each of the comments. All the changes are shown in red font in the “**Revised manuscript with Track Changes**” file for reviewer’s understanding the previous and changes sentences. A **“Manuscript”** of revised paper without track changes also attached here with for easy check.

**Journal requirements:**

1. **Please ensure that your manuscript meets PLOS ONE's style requirements, including those for file naming.**

Response/Action: Thanks to the editor for this nice suggestion. Following his comment, we have corrected the format of our manuscript according to PLOS ONE’s requirements. We have also changed the file name here.

**Before revision:**

File name: Manuscript for Plos one

**After revision:**

File name: Manuscript. Please go through the file “[Revised Manuscript with Track Changes.docx](Revised%20Manuscript%20with%20Track%20Changes.docx)”

2. **Please state what role the funders took in the study.  If the funders had no role, please state: "The funders had no role in study design, data collection and analysis, decision to publish, or preparation of the manuscript."**

**Response/Action: Thank you for this comment**

**Before revision:**

“All financial support for this work was provided by Bangladesh Council of Scientific and Industrial Research (BCSIR), Dhaka, Bangladesh.”

**After revision:**

All financial support for this work was provided by Bangladesh Council of Scientific and Industrial Research (BCSIR), Dhaka, Bangladesh. As it is a Government organization and I am an employee here, BCSIR has provided me with fund but it has no role in study design, data collection and analysis, decision to publish or preparation of manuscript. This statement has amended on submission form as well as in “cover letter”.

Reviewer’s comments:

1. **Is the manuscript technically sound, and do the data support the conclusions?**

**Reviewer**: Partly

Response/Action: Thank you for your comment. Based on reviewers’ comment, we have modified the conclusion based on the data so that it can support the research. All works have done in triplicate set with appropriate control where necessary.

**Before revision:**

**Conclusion**-This study concentrated on the isolation, characterization, and evaluation of antimicrobial activity of prodigiosin synthesized by newly isolated bacterium from the soil of BCSIR Rajshahi Laboratories, Bangladesh. This investigation identified the isolated bacteria as Serratia sp. BRL41 harboring the prodigiosin biosynthetic gene cluster. Prodigiosin synthesis by isolate BRL41 attained its maxima at 30˚C temperature, pH 7.5 after 96 hours of incubation. It appeared as a potential biological agent inhibiting bacterial growth and biofilm formation by lysing bacterial cell walls. Thus, this study may aid the development of an antimicrobial drugs from prodigiosin to combat bacterial infection. Additionally, it can encourage researchers to work with the prodigiosin in detail to determine its mode of action.

After revision:

Conclusion- In a nutshell, our study demonstrates the production, optimization and characterization of prodigiosin by a newly isolated local *Serratia sp.* along with evaluation of its some biological and pharmaceutical characteristics. Prodigiosin has found antagonist against Gram-positive and Gram-negative bacteria where the possible mechanism can be the cell wall disruption or production of reactive oxygen species. Prodigiosin also reduces biofilm formation possibly by modulating virulence gene expression, aggregation, hydrophobicity, acid production, acid tolerance. Increase of intracellular glutathione is a proof towards prodigiosin’s antioxidant activity. These findings suggest that prodigiosin can be used for the development of antimicrobial drug that can combat bacterial infection. In addition, identification of core and regulatory genes in BGC can work as a fundament for future improvement and modification of prodigiosin production by BRL41.Besides, further studies are required for better understanding of molecular mechanisms underlying the inhibitory effect of prodigiosin against pathogenic bacteria.

2. **Has the statistical analysis been performed appropriately and rigorously?**

**Reviewer**: No

**Response/ Action:** Thank you for reviewer’s great comment. Following reviewers’ comment, we have analyzed all data using IBM SPSSS statistics 22 software. All data are expressed as mean± standard deviation where p value was calculated at 95% significant level. Please go through the file “[Revised Manuscript with Track Changes.docx](Revised%20Manuscript%20with%20Track%20Changes.docx)”

**Before revision:**

**Statistical analysis: No information available**

**After revision:**

Statistical analysis was performed using IBM SPSSS statistics 22 software. All data were expressed as mean ± standard deviation. One-way ANOVA followed by Tukey test was conducted for comparing means of zone of inhibition. p value < 0.05 were considered to be statistically significant at 95% confidence level.

3. **Have the authors made all data underlying the findings in their manuscript fully available?**

**Reviewer**: Yes

**Response/Action:** Thank you for reviewer’s great comment.

4. **Is the manuscript presented in an intelligible fashion and written in standard English?**

**Reviewer:** No

**Response/Action**: With some data rearrangement and change of language, we have tried our best to present the manuscript in an intelligible fashion with simple English that can become easily understandable to all. Please have a look at file “[Revised Manuscript with Track Changes.docx](Revised%20Manuscript%20with%20Track%20Changes.docx)”

5. **This work lacks originality as most findings were described elsewhere (Production of prodigiosin by S marcescens. it's inhibitory and antibiofilm effects. etc). Other investigations should be added if the authors want to give advanced findings. (for example, discovery of new biological or pharmacological effects). In addition, the authors should rearrange, and consider rewriting in order to rise originality of the work, consider Figure relevance for publication and do statistics for comparison of significant different results.**

**Response/Action**: Thank you for reviewer’s comment and suggestions. Many works have been conducted on prodigiosin in different parts of the world but in Bangladesh this work has done for the first time with a new isolate obtained from Barind soil (a special type of native soil in Rajshahi region of Bangladesh).Following the suggestion of reviewer we have analyzed the zone of inhibition data in a different way to show whether change of prodigiosin concentrations affect antimicrobial activity or not. In addition, we have explored a new pharmaceutical activity of prodigiosin i.e cellular reduced glutathione level (GSH) measurement which is, to the best of our knowledge, the first work with prodigiosin. Besides, we have changed figure 9 (Protein release assay) and presented it in a more acceptable fashion.

**Before revision:**

| **Bacteria** | **Diameter of Inhibition mm** | | | | |
| --- | --- | --- | --- | --- | --- |
|  | Positive Control | Negative Control | Prodigiosin | | |
|  | Chloramphenicol  (30µg/mL) | DMSO | 500 µg/mL | 250  µg /mL | 125  µg /mL |
| *E coli* | 26±1.00 | 0 | 21±0.60 | 17±0.69 | No zone |
| *Staphylococcus aureus* | 28±0.58 | 0 | 19±0.56 | 14±0.76 | 9±0.00 |
| *Listeria monocytogens* ATCC-3193 | 28.67±0.58 | 0 | 19±0.10 | 13±0.34 | 10±1.15 |
| *Pseudomonas aeruginosa* ATCC- 9027 | 26±0.58 | 0 | 25±0.25 | 17±0.20 | No zone |
| *Salmonella enterica* ATCC-10708 | 25±0.58 | 0 | 20±0.09 | 8±0.75 | No zone |

**After revision:**

|  | **Concentration** | **Tested bacteria** | | | | | |
| --- | --- | --- | --- | --- | --- | --- | --- |
|  | **(µg/ml)** | | ***E. coli*** | ***Staphylococcus aureus*** | ***Listeria monocytogens* ATCC-3193** | ***Pseudomonas aeruginosa* ATCC- 9027** | ***Salmonella enterica* ATCC-10708** |
| Prodigiosin | 500 | 25.61 ± 0.15^a^ | | 18.66 ± 0.0.33^b^ | 18.56 ± 0.22^b^ | 24.73 ± 0.15^b^ | 19.60 ± 0.05^b^ |
|  | 250 | 16.89 ± 0.40^b^ | | 14.00 ± 0.45^c^ | 13.00 ± 0.20^c^ | 16.61 ± 0.11^c^ | 7.77 ± 0.39^b^ |
|  | 125 | No zone | | 9.00 ± 0.00^d^ | 9.00 ± 0.00^d^ | No zone | No zone |
|  | +ve (control) | 26.00 ± 0.58^a^ | | 27.67 ± 0.33^a^ | 27.67 ± 0.33^a^ | 26.33 ± 0.33^a^ | 25.00 ± 0.00^a^ |

**Before revision:**

No data about GSH content measurement of blood cell after exposing on prodigiosin

**After revision:**

## **Prodigiosin increased cellular GSH level**

Over the range of 125-500 µg/ml, prodigiosin significantly increased (p< 0.05) reduced glutathione level (GSH) in cell at 250 and 500 µg/ml concentration **“Fig 8”.** Prodigiosin at these concentrations stimulated glutathione synthesis that proved its efficacy as an antioxidation stimulating agent

**Fig 8: Effect of prodigiosin on increasing GSH level of blood cell.Data are presented as mean±standard deviation. *p value < 0.05 represents significant difference with control**

**Before revision:**

**Fig 9: Protein release from prodigiosin (500 µg/mL) treated and untreated A) Gram-positive and B) Gram-negative cell**

**After revision:**

**Fig 9: Protein release from prodigiosin (500 µg/mL) treated and untreated A) Gram-positive and B) Gram-negative cell**

**Other remarks:**

**Line19: E. coli: Please to write complete name of the species
What is type strains of “Staphylococcus aureus”.**

**Action: Amended**

**Before revision:**
*E.coli*, *Staphylococcus aureus*,

**After revision:**

***Escherichia coli,*** *Staphylococcus aureus* (environmental isolate)

**Line 22-24: finding not clear, please to support briefly by arguments**

**Action:** Thank you for this comment. For better understanding and to support our arguments we have tried to explain the work more clearly than before.

**Before revision:**

Furthermore, the increase of protein concentration in prodigiosin treated bacterial suspension was an evidence of cell lysis resulting in protein release from cells.

**After revision:**

Cell wall as one of the sites of action of prodigiosin was manifested by the increase of protein concentration on prodigiosin treated cell suspension that was resulted from protein leakage by damaged cell wall over time.

**Line 79-84: Please to formulate clearly ideas specifically relationships and outcomes regarding BGC and prodigiosin. Please to give clear originality of the work. Please to focus on outcomes and significance of your results rather than describing methods. In the absence of originality, the MS is not suitable to be published in the PLOS journal**

**Action: We have re-written this section with adding more relevant informations.**

**Before revision:**

So, the study observed the behavior of prodigiosin towards both Gram-positive and Gram-negative bacterial pathogens. Firstly, *Serratia* sp. BRL41 with prodigiosin-producing capability was isolated from soil in BCSIR, Rajshahi, Bangladesh. Prodigiosin was produced and extracted using solvent extraction method followed by its identification using Thin-layered Chromatography (TLC), UV-spectroscopy, Fourier Transform Infrared Spectroscopy (FTIR), and pH dependent color change. This study also focused on finding the optimum conditions for bacterial prodigiosin biosynthesis and extraction. Another aspect of this study was to utilize the whole genome sequence data of the isolate to identify the Biosynthetic Gene Clusters (BGC) associated with secondary metabolite production. Antimicrobial activity of prodigiosin was tested by agar well diffusion method and minimum inhibitory concentration (MIC) and minimum bactericidal concentration (MBC) assay. Biofilm formation inhibition and protein release capability of prodigiosin were also explored here. The outcomes give an evidence of prodigiosin’s bactericidal efficacy that facilitates its survival at competitive environment as well as explore a potential bacterial metabolite that can be used as alternative to antibiotic.

**After revision:**

So, the study addressed the bacteriostatic/bactericidal behavior of prodigiosin towards both Gram-positive and Gram-negative bacterial pathogens. *Serratia* sp. BRL41 is the first reported prodigiosin-producing bacteria in Bangladesh isolated from barhind soil. Prior to checking antimicrobial activity,prodigiosin was identified and characterized. Another aspect of this study was to utilize the whole genome sequence data of the isolate to identify the Biosynthetic Gene Clusters (BGC) associated with secondary metabolite biosynthesis that facilitated the understanding of prodigiosin biosynthesis and regulation mechanism with the identification of core biosynthetic gene in BRL41. For the first time, we have investigated membrane integrity of Gram-positive and Gram-negative bacterial cell upon treated with prodigiosin from *Serratia marcescens* as well as role of prodigiosin as an anti-oxidant by measuring intracellular reduced glutathione level (GSH) of human blood cell. In a nutshell, in search of new antimicrobial molecules, prodigiosin’s biological and pharmaceutical efficacies provides evidence of its potency as an alternative to antibiotics.

**Line 104: Give manufacturer name**

**Action:** Manufacturer’s name of Wizard® Genomic DNA Purification Kit has added.

**Before revision:**

DNA from the BRL41 isolate was extracted using Wizard® Genomic DNA Purification Kit and whole genome sequencing was done by Illumina MiniSeq platform.

**After revision:**

DNA from the BRL41 isolate was extracted using Wizard® Genomic DNA Purification Kit (Promega, catalog no. PR-A1120) and whole genome sequencing was done by Illumina MiniSeq platform.

**Line107: give reference for “gene assembly by Shovill pipeline ».**

**Action: Reference of Shovill pipeline has added here.**

**Before revision:**

Library preparation was conducted as per the manufacturer’s protocol. After obtaining the raw data, the assembly of the genome was performed using the Shovill pipeline. This genome assembler utilized SPAdes as its core and is specific for bacterial isolated genomes from Illumina paired end reads.

**After revision:**

After obtaining the raw data, the assembly of the genome was performed using the Shovill pipeline (*GitHub - Tseemann/Shovill: Assemble Bacterial Isolate Genomes from Illumina Paired-End Reads*, n.d.)This genome assembler utilized SPAdes as its core and is specific for bacterial isolated genomes from Illumina paired end reads.

**Line 146-147: please to rewrite**

Action: The sentence has corrected. In addition, we have re-written this section.

**Before revision:**

Optimum temperature for pigment biosynthesis was determined by inoculating nutrient broth with bacteria was incubating at 25°C, 28°C, 30°C, 37°C temperatures. After 96 hours of incubation, the color intensity was measured spectrophotometrically at 537 nm and the result was recorded. One of the crucial factors for pigment production, the pH, was fixed by allowing BRL41 to grow at different pH nutrient broth ranged from 6.00 to 8.50. Pigment production at each pH medium was quantified to determine the optimum pH for maximum prodigiosin production.

**After revision:**

To find out optimum conditions for maximum prodigiosin production by Smi41, some important parameters like incubation time (24, 48, 72, 96 hour), temperature (25, 28, 30, 37˚ C), pH (5.00 to 8.5) were checked using nutrient broth as basal media. After each treatment color intensity was measured spectrophotometrically at 537 nm where the highest absorbance signified the highest pigment production on that particular condition.

**Please to add references for techniques in M&M.**

**Action:** Thank you for this great suggestion. We have added references for all the techniques used to conduct this work**.**

**Before revision:**

**Phylogenetic and Biosynthetic Gene Cluster (BGC) analysis**

DNA from the BRL41 isolate was extracted using Wizard® Genomic DNA Purification Kit and whole genome sequencing was done by Illumina MiniSeq platform. Library preparation was conducted as per the manufacturer’s protocol. After obtaining the raw data, the assembly of the genome was performed using the Shovill pipeline. This genome assembler utilized SPAdes as its core and is specific for bacterial isolated genomes from Illumina paired end reads. Phylogenetic analysis was performed using Type (Strain) Genome Server (TYGS) using maximum-likelihood method (21). The phylogenetic tree was visualized using iTOL(22). Biosynthetic gene clusters responsible for the secondary metabolite prodigiosin production were identified using antiSMASH (23). This identified bacterial isolate was used to conduct further studies.

**Determination of Minimum Inhibitory Concentration (MIC) and Minimum Bactericidal Concentration (MBC)**

MIC of prodigiosin was assessed against these five species of bacteria by dilution method in test tubes. Firstly, the optical density of 24 hours culture of each bacterium was adjusted to 0.4-0.6. Then a solution of prodigiosin with a final concentration of 500µg/mL was prepared using 10% dimethyl sulfoxide (DMSO). 1ml of prodigiosin was added in the 1st tube and then was serially diluted to obtain concentrations ranging from 500µg/mL to 0.24µg/mL. Tube containing culture broth and inoculum represented a positive control whereas a tube with culture broth, inoculum, and antibiotic chloramphenicol was used as a negative control. These were incubated at 30°C overnight. Then the growth pattern of bacteria was observed to determine the minimum inhibitory concentration based on the absence of visible growth of each bacterium. MBC was determined by spreading 10 µL of culture from each tube on nutrient agar plate to find out the concentration that caused the complete death of bacterial cells. Concentration for which no bacterial growth was observed on the plate was considered as MBC.

**Anti-biofilm activity of prodigiosin**

The ability of prodigiosin to inhibit biofilm formation of pathogenic bacteria was evaluated by crystal violet (CV) assay in 96 well microtiter plates. A serial dilution of prodigiosin was made using 10% DMSO in a microtiter plate and bacterial culture was then added to every well to make a final volume of 100 µL. For every bacterium, nutrient broth represented blank, whereas a mixture of nutrient broth, DMSO, and 10 µL of bacteria was used as control. After 48 hours of incubation, each well was washed twice with 1×PBS solution followed by the addition of 200µL of 99% methanol. It was kept for 20 minutes and then the methanol was discarded. After drying the plate for 15 minutes, 200 µL of 0.1% crystal violet was added to each well and kept at room temperature for 5 minutes so that it could stain the bacterial cell. Crystal violet was drained out and the plate was dried completely. Finally, to solubilize the crystal violet attached to the bacterial cell, 95% ethanol was added and mixed. Then the optical density was measured at 450nm using a microtiter plate reader. Biofilm inhibition % was calculated using the following formula (25)

$$\frac{{(OD}_{\mathrm{control}}-\mathrm{OD}_{\mathrm{treated}})}{\mathrm{OD}_{\mathrm{control}}}\times100$$

**After revision:**

## **Phylogenetic and Biosynthetic Gene Cluster (BGC) analysis**

DNA from the BRL41 isolate was extracted using Wizard® Genomic DNA Purification Kit (Promega, catalog no. PR-A1120) and whole genome sequencing was done by Illumina MiniSeq platform. Library preparation was conducted as per the manufacturer’s protocol. After obtaining the raw data, the assembly of the genome was performed using the Shovill pipeline (21).This genome assembler utilized SPAdes as its core and is specific for bacterial isolated genomes from Illumina paired end reads. Phylogenetic analysis was performed by Type (Strain) Genome Server (TYGS) using maximum-likelihood method (22). The phylogenetic tree was visualized using iTOL(23). Biosynthetic gene clusters responsible for the secondary metabolite prodigiosin production were identified using antiSMASH (24). This identified bacterial isolate was used to conduct further studies.

## **Determination of Minimum Inhibitory Concentration (MIC) and Minimum Bactericidal Concentration (MBC)**

MIC of prodigiosin was assessed against these five species of bacteria by macro dilution method in test tubes (26). Firstly, the optical density of 24 hours culture of each bacterium was adjusted to 0.4-0.6. Then a solution of prodigiosin with a final concentration of 500µg/mL was prepared using 10% dimethyl sulfoxide (DMSO). 1ml of prodigiosin was added in the 1st tube and then was serially diluted to obtain concentrations ranging from 500µg/mL to 0.24µg/mL. Tube containing culture broth and inoculum represented a positive control whereas a tube with culture broth, inoculum, and antibiotic chloramphenicol was used as a negative control. These were incubated at 30°C overnight. Then the growth pattern of bacteria was observed to determine the minimum inhibitory concentration based on the absence of visible growth of each bacterium. MBC was determined by spreading 10 µL of culture from each tube on nutrient agar plate to find out the concentration that caused the complete death of bacterial cells. Concentration for which no bacterial growth was observed on the plate was considered as MBC.

## **Anti-biofilm activity of prodigiosin**

The ability of prodigiosin to inhibit biofilm formation of pathogenic bacteria was evaluated by crystal violet (CV) assay in 96 well microtiter plates (27). A serial dilution of prodigiosin was made using 10% DMSO in a microtiter plate and bacterial culture was then added to every well to make a final volume of 100 µL. For every bacterium, nutrient broth represented blank, whereas a mixture of nutrient broth, DMSO, and 10 µL of bacteria was used as control. After 48 hours of incubation, each well was washed twice with 1×PBS solution followed by the addition of 200µL of 99% methanol. It was kept for 20 minutes and then the methanol was discarded. After drying the plate for 15 minutes, 200 µL of 0.1% crystal violet was added to each well and kept at room temperature for 5 minutes so that it could stain the bacterial cell. Crystal violet was drained out and the plate was dried completely. Finally, to solubilize the crystal violet attached to the bacterial cell, 95% ethanol was added and mixed. Then the optical density was measured at 450nm using a microtiter plate reader. Biofilm inhibition % was calculated using the following formula (28)

$$\frac{{(OD}_{\mathrm{control}}-\mathrm{OD}_{\mathrm{treated}})}{\mathrm{OD}_{\mathrm{control}}}\times100$$

## **Antioxidant assay on by estimating reduced glutathione (GSH) content in cell**

This test was performed following the methodology described by (29) with some modification. In brief, Prodigiosin of varying concentrations (125,250,500 µg/ml) were taken in three separate falcon tube and erythrocyte suspension was added and mixed by gentle inversion. Erythrocytes with PBS was served as control and all the tubes were incubated at 37˚C for 24 hours. After centrifugation of reaction mixtures, water addition facilitated cell lysis followed by addition of metaphosphoric acid that resulted in precipitation of cell lysate. Following centrifugation both control and treatment tubes were treated with 0.01 M sodium phosphate buffer (pH 7.5), 5 mM EDTA, 10 mM 5,5-dithio-bio-nitrobenzoic acid(DTNB), 2mM NADPH, 100 U/ml glutathione reductase to reach a final volume 1ml.Absorbence was taken at 412 nm against blank.

**Line 286: Write “s” in “staphylococcus » in capital letter**

**Action**: In Staphylococcus small “s”has replaced with capital “S”

**Before revision:**

A higher MIC (> 10µg/mL) was required to inhibit the growth of *E. coli, staphylococcus aureus*, and *Listeria monocytogenes* ATCC-3193 compared to *Pseudomonas aeruginosa* ATCC-9027, and *Salmonella enterica* ATCC-10708.

**After revision:**

A higher MIC (> 10µg/mL) was required to inhibit the growth of *E. coli, Staphylococcus aureus*, and *Listeria monocytogenes* ATCC-3193 compared to *Pseudomonas aeruginosa* ATCC-9027, and *Salmonella enterica* ATCC-10708.

**Line 227: please write “sakeunsis” in italic**

**Action:** The word “sakeunsis”has changed to italic

**Before revision:**

Sequence alignment and construction of the phylogenetic tree revealed that the isolate *Serratia sp.* BRL41 is very closely related to strain *Serratia marcescens* subsp., sakeunsis KCTC 42172, and *Serratia marcescens* ATCC 13880

**After revision:**

Sequence alignment and construction of the phylogenetic tree revealed that the isolate *Serratia sp.* BRL41 is very closely related to strain *Serratia marcescens* subsp., *sakeunsis* KCTC 42172, and *Serratia marcescens* ATCC 13880

**Figure 1: Gram reaction of isolate BRL41 There is no need for this figure, as simple description in the text is sufficient**

**Action:** Based on the comment of reviewer’s**,** Gram reaction figure of isolate BRL41 has delated from revised manuscript.

**Figure 2: There is no need for this display, or to move to Appendix.**

**Action:** Thank you for your suggestion**.** Figure 2 of original manuscript has been delated in revised one

**Figure 5: There is no need for this figure (part showing color change), as simple description in the text is sufficient**

**Action:** Thanks for reviewer’s comment. Based on it, the figure has corrected by deleting color changing part and kept only the TLC section**.**

**Before action:**

**After action:**
