# Supplementary material for: In silico exploration of Serratia sp. BRL41 genome for detecting prodigiosin Biosynthetic Gene Cluster (BGC) and in vitro antimicrobial activity assessment of secreted prodigiosin
Source: PLoS One. 2023 Nov 15;18(11):e0294054. doi: 10.1371/journal.pone.0294054 (PMC10651056; doi:10.1371/journal.pone.0294054)
Supplement: Attachment — Submitted filename: Response to Reviewers.docx [file pone.0294054.s002.docx]

**Response to Reviewers**

# **Response to Reviewers**

## **Manuscript PONE- D-23-01982R2**

On behalf of co-authors, I would like to thanks the reviewers who gave made few comments on the manuscript PONE-D-23-01982R2 for improvement. We have addressed all the comments raised by the reviewers and revised the manuscript accordingly. Please find our changes for each of the comments in this disposition. All the changes are shown in red font in the “**Revised manuscript with Track Changes**” file for reviewer’s understanding the previous and changes sentences. A **“Manuscript”** of revised paper without track changes also attached here with for easy check.

**Reviewers comments:**

1. If the authors have adequately addressed your comments raised in a previous round of review and you feel that this manuscript is now acceptable for publication, you may indicate that here to bypass the “Comments to the Author” section, enter your conflict of interest statement in the “Confidential to Editor” section, and submit your "Accept" recommendation.

Reviewer #2: All comments have been addressed

Reviewer #3: All comments have been addressed

Response/Action: Thank you for all reviewers’ great comment

2. Is the manuscript technically sound, and do the data support the conclusions?

Reviewer #2: Yes

Reviewer #3: Yes

Response/Action: Thank you for reviewers’ positive response

3. Has the statistical analysis been performed appropriately and rigorously?

Reviewer #2: Yes

Reviewer #3: Yes

Response/Action: Thank you for all reviewers’ great comment

4. Have the authors made all data underlying the findings in their manuscript fully available?

Reviewer #2: Yes

Reviewer #3: Yes

Response/Action: Thank you for reviewers’ positive response

5. Is the manuscript presented in an intelligible fashion and written in standard English?

   Reviewer #2: Yes

Reviewer #3: Yes

Response/Action: Thank you for all reviewers’ great comment

6. **Review Comments to the Author**

**Reviewer #2:**

1. The manuscript has been significantly improved. However, the membrane integrity part still suffers from lack of proper justification. Protein leakage assay is acceptable but it cannot justify the loss of membrane integrity, unless it is linked to direct evidences of membrane damage. Authors should link their findings to those of previous studies to justify their results. Please refer to the findings of Lependa et al. (2015), Kimyon et al. (2016), Suryawanshi et al. (2017), Hazarika et al. (2021), and Ravindran et al. (2020).

Response/ action: Thank you for reviewer’s comments. Based on these, we have made some corrections in discussion section regarding membrane integrity to make the highest alignment with findings and in future we will definitely find out direct evidence to support justification.

All above mentioned papers have been cited with reference number 41,17,51,49, and 33 respectively.

Before revision: When introduced into cell, prodigiosin immediately inserts into the cell membrane and disrupt its integrity, leading to leakage of protein and ATP (55). Herein, after prodigiosin application, measurement of cell free protein in *Listeria monocytogenes* ATCC-10708 and *E. coli* culture broth also support this finding. In both Gram-positive and Gram-negative culture, protein concentrations followed an upward trend with time and then decreased slightly. This could be due to the production of surfactant by competent cells that formed strong complexes with the Bradford reagent, making it unavailable for protein binding and interfering with the result

After revision: . For prodigiosin, a hydrophobic stressor is responsible to disrupt cellular plasma membrane via a chaotropicity-mediated-mode-of-action. Therefore, when introduced into cell, prodigiosin immediately inserts into the cell membrane and disrupt its integrity, leading to leakage of protein and ATP (51). Herein, after prodigiosin application, measurement of cell free protein in *Listeria monocytogenes* ATCC-10708 and *E. coli* culture broth also support this finding. In both Gram-positive and Gram-negative culture, protein concentrations followed an upward trend with time and then decreased slightly. This could be due to the production of surfactant by competent cells that formed strong complexes with the Bradford reagent, making it unavailable for protein binding and interfering with the result (52). A further investigation is required to observe the interaction between pigment and membrane to support this evidence.

2. All the references need to be synchronized, especially those having an internet link

Response/ action: Thank you so much for this comment. All the references have synchronized in the same format. Please have a look at the “Reference section” of the manuscript.

**Reviewer #3:**

 Authors have taken into considerations all my comments in the revised manuscript. I have just noticed a bad resolution of figures. It would be better to improve during the publication process.

Response/ action: Thank you so much for the comment. We have improved the quality of our figures to fit it for publication in “PLOS ONE”
